# AA amyloid fibrils from diseased tissue are structurally different from in vitro formed SAA fibrils

**DOI:** 10.1038/s41467-021-21129-z

**Published:** 2021-02-12

**Authors:** Akanksha Bansal, Matthias Schmidt, Matthies Rennegarbe, Christian Haupt, Falk Liberta, Sabrina Stecher, Ioana Puscalau-Girtu, Alexander Biedermann, Marcus Fändrich

**Affiliations:** Institute of Protein Biochemistry, Ulm University, Ulm, Germany

**Keywords:** Protein aggregation, Cryoelectron microscopy

## Abstract

Systemic AA amyloidosis is a world-wide occurring protein misfolding disease of humans and animals. It arises from the formation of amyloid fibrils from serum amyloid A (SAA) protein. Using cryo electron microscopy we here show that amyloid fibrils which were purified from AA amyloidotic mice are structurally different from fibrils formed from recombinant SAA protein in vitro. Ex vivo amyloid fibrils consist of fibril proteins that contain more residues within their ordered parts and possess a higher β-sheet content than in vitro fibril proteins. They are also more resistant to proteolysis than their in vitro formed counterparts. These data suggest that pathogenic amyloid fibrils may originate from proteolytic selection, allowing specific fibril morphologies to proliferate and to cause damage to the surrounding tissue.

## Abstract (web summary)

Systemic AA amyloidosis is a protein misfolding disease caused by the formation of amyloid fibrils from serum amyloid A (SAA) protein. Here, the authors present the cryo-EM structures of AA amyloid fibrils isolated from mouse tissue and in vitro formed fibrils, which differ in their structures and they also show that the ex vivo fibrils are more resistant to proteolysis than the in vitro fibrils and propose that pathogenic amyloid fibrils might originate from proteolytic selection.

## Introduction

Much of our understanding of the structure, formation and biological activity of disease-associated amyloid fibrils has been obtained from the analysis of amyloid fibrils that were formed in vitro^1,2^. In vitro fibrils reproduce the generic structural features of amyloid fibrils, such as their linear morphology, cross-β structure and affinity for Congo red and thioflavin T dyes^3^, but an increasing number of recent studies found fibrils from patient tissue to be structurally different from in vitro fibrils from the same precursor protein. Examples hereof are the immunoglobulin light chain-derived fibrils in systemic AL amyloidosis^4,5^, the Aβ-derived fibrils in Alzheimer’s disease^6^, tau-derived fibrils in neurodegenerative diseases^7^, or the α-synuclein-derived fibrils in multiple system atrophy^8^. Hence, out of the spectrum of fibril morphologies, that a polypeptide chain is able to adopt, only some morphologies are associated with disease, raising the question what might determine their pathological relevance?

Several studies implied that pathogenicity and disease variant are correlated with specific fibril morphologies. Consistent fibril morphologies were found at different deposition sites within the same patient or animal^4^. Consistent fibril morphologies were also found if fibrils were extracted from humans or animals that suffered from the same disease variant and expressed the same allelic variant of the fibril precursor protein^4,6,8–11^. Conversely, different morphologies and biochemical characteristics of the fibrils are associated with different disease variants^12–14^. Hence, it would constitute a major step in understanding if the formation of the pathogenically relevant amyloid fibril morphologies could be explained.

In this research we have focused on the amyloid fibrils in systemic AA amyloidosis. This potentially fatal disease^15^ was abundant in Western countries until the mid of the 20th century, as suggested by retrospective analyses of historical medical collections^16^, and it probably still is until today in other parts of the world^17^. The disease is unique amongst all protein misfolding diseases because it occurs not only in humans but also in many birds and mammalian species^18^. Hence, it provides the possibility to study disease mechanisms in animals, and in particular in mice.

Mice express several SAA family members, similar to humans, but only SAA1.1 is a major fibril precursor protein, corresponding to the prime role of SAA1.1 in Caucasian populations^19^. SAA1.1 is an acute phase protein that becomes strongly upregulated during inflammation. Chronic inflammatory conditions, such as tuberculosis, leprosy and rheumatoid arthritis, are risk factors for the development of systemic AA amyloidosis^17,20^. We here report an analysis of the structure of SAA1.1-derived amyloid fibrils from AA amyloidotic mice or formed in vitro from recombinant murine SAA1.1 protein.

## Results

### Polymorphism of AA amyloid fibrils from murine tissue

**Fig. 1:**
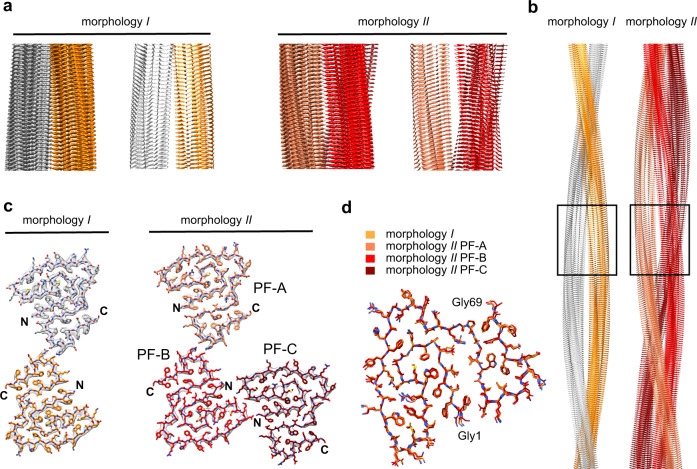
Cryo-EM structures of AA amyloid fibrils purified from diseased tissue. **a** Side views of fibril morphologies I and II: corresponding segments of the 3D maps and models, shown as ribbon diagrams. **b** Models of fibrils in side view. The segment shown in **a** is boxed. **c** Cross-sectional layers of fibril morphologies I and II. The 3D maps (gray surfaces) are superimposed with the models, shown as sticks. **d** Alignment of the fibril proteins of morphology I and II (PF-A to PF-C) as indicated in the figure. The data of morphology I is taken from a previously deposited data (PDB 6DSO: https://doi.org/10.2210/pdb6dso/pdb)^50^.

Purified AA amyloid fibrils from mice are polymorphic^4,21^. They contain a dominant fibril morphology (morphology I, Supplementary Fig. 1a) that accounts for 95% of the fibrils^22^. The second most abundant fibril morphology (morphology II) represents the majority of the remaining fibrils. Morphology II presents a left-hand twist as demonstrated by platinum side shadowing and transmission electron microscopy (TEM) (Supplementary Fig. 1b). Using cryo-electron microscopy (cryo-EM) we obtained the structure of morphology II, complementing our previous structure of morphology I^22^. The 3D map of morphology II achieved a spatial resolution of 3.5 Å (Fig. 1a, b, Supplementary Fig. 1c, Supplementary Table 2), based on the 0.143 Fourier shell correlation (FSC) criterion (Supplementary Fig. 1d). The resolution shows local variations in the fibril cross-section (Supplementary Fig. 1e). Interpretation of the 3D map led to a molecular model (Supplementary Table 3) that fits well to the reconstructed density (Fig. 1c), 2D class averages and power spectra (Supplementary Fig. 2).

Morphology II is polar, C1 symmetrical and consists of three protofilaments (PFs) (Fig. 1c). The PFs are referred to as PF-A, PF-B, and PF-C. The arrangement of PF-A and B in our model is relatively symmetrical and similar to morphology I (Supplementary Fig. 3a, b). The PF-PF interface is formed in both cases by reciprocal ion pairs between residues Asp59 and Arg61. The interactions between PF-B and C also involve complementary charged residues. There are reciprocal interactions between Asp22 and Lys24, as well as a contact of Gly1 (α-amino group) from PF-C with Glu25 (side chain) in PF-B (Supplementary Fig. 3a). That is, the packing between PF-B and PF-C is more asymmetrical than that of PF-A and PF-B. Alignment of the fibril proteins reveals only small, if any, conformational differences between the protein folds that construct fibril morphologies I and II (Fig. 1d). Their main difference is the PF number.

### Polymorphism of the in vitro formed SAA1.1 fibrils

In vitro formed fibrils are also polymorphic and contain one major fibril morphology (morphology i, Supplementary Fig. 4a). This fibril corresponds to 75% of the fibrils present in solution and is considerably thinner than the other analyzed fibril morphology, termed here morphology ii (Supplementary Fig. 4a). The latter represents only 7% of the fibrils. Both fibrils are left-hand twisted (Supplementary Fig. 4b). Cryo-EM reconstructions achieved spatial resolutions of 2.73 Å for morphology i and 2.95 Å for morphology ii (Supplementary Fig. 4d, Supplementary Table 2). The correlation of the structural models (Supplementary Table 3) with the reconstructed 3D maps, 2D class averages and power spectra is shown in Supplementary Fig. 5.

**Fig. 2:**
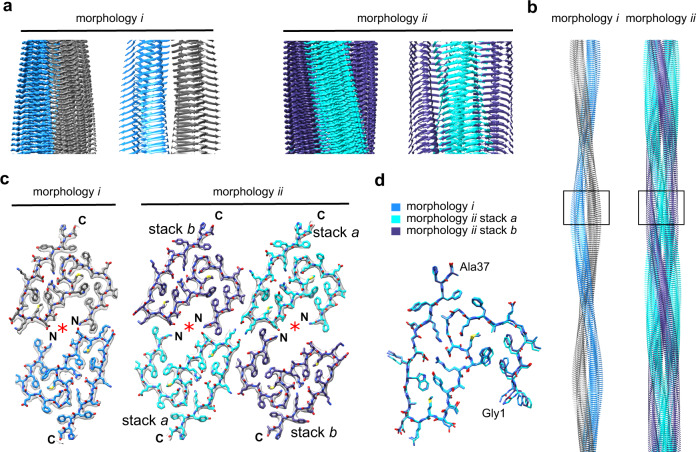
Cryo-EM structures of amyloid fibrils from recombinant SAA1.1 protein in vitro. **a** Side views of fibril morphologies i and ii: corresponding sections of the 3D maps and models, shown as ribbon diagrams. **b** Models of fibrils in side view. The segment shown in **a** is boxed. **c** Cross-sectional layers of fibril morphologies i and ii. The 3D maps (gray surfaces) are superimposed with the models, shown as sticks. Red asterisk: cavity of the structure. **d** Alignment of the fibril proteins of morphology i and ii (stacks *a* and *b*) as indicated in the figure.

Morphology i is polar and presents a pseudo 2_1_-screw symmetry (Fig. 2a, b). A single PF consists of two structurally equal stacks of fibril proteins that are arranged such that residues Gly1 and Phe2 from one stack interact with residues Asp22-Lys24 in the other stack (Supplementary Fig. 6). The Gly1 α-amino group is in the vicinity of the Asp22 side chain, while Phe2 inserts into a small pocket formed by Asp22-Lys24 main chain and side chain atoms. The PF encloses a large internal cavity that appears water filled as it is lined with polar (Thr21) and charged (Gly1, Asp22) amino acid residues (Fig. 2c).

Morphology ii is also polar but presents C2 symmetry (Fig. 2a, b). It consists of two PFs that both exhibit the structure of the morphology i PF. Both fibril morphologies contain an identical protein fold (Fig. 2d). In contrast to morphology i, however, morphology ii PFs contains two non-equal protein stacks, termed here stacks *a* and *b* (Fig. 2c). They differ in their relative position in the fibril structure and at the interface between the two PFs. This interface involves, similar to the ex vivo fibrils, complementary charged amino acids. Lys24 (stack *a*) in one PF is packed against Glu25 (stack *a*) in the other PF. Glu8 (stack *b*) in one PF is opposite to Lys29 (stack *a*) in the other PF (Supplementary Fig. 6).

In conclusion, both in vitro formed fibril morphologies contain the same fibril protein fold and differ in the PF number. No cryo-EM reconstructions could be obtained for any of the remaining fibril morphologies of this sample, but analysis of their structural parameters in the cryo micrographs implies that they do not correspond to the ex vivo fibril morphologies described above.

### Ex vivo fibrils structurally vary from in vitro fibrils

**Fig. 3:**
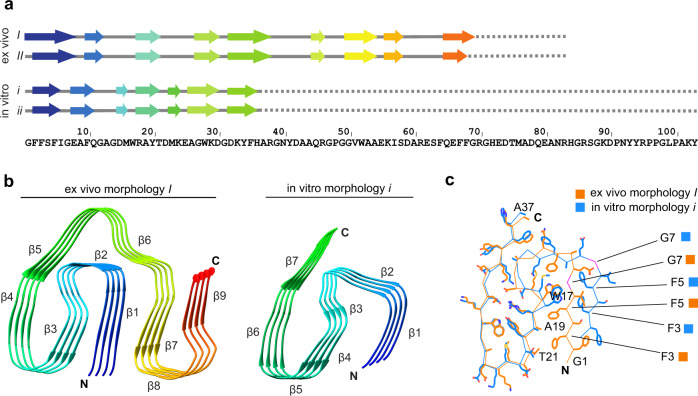
Fibril protein conformation in in vitro and ex vivo fibrils. **a** Sequence of SAA1.1 indicating the location of the β-strands. **b** Ribbon diagrams of fibril protein stacks (ex vivo fibril morphology I and in vitro fibril morphology i) colored in rainbow palette. **c** Alignment of residues 1–37 of the ex vivo (orange) and in vitro fibril protein structure (blue).

In vitro and ex vivo fibrils possess parallel cross-β sheets (Fig. 3a, b) but differ in their overall topologies and fibril protein folds. The fibril protein of ex vivo fibrils adopts a stable conformation at residues Gly1-Gly69. It is compact and consists of nine β-strands at residues Phe2-Glu8, Phe10-Gly12, Arg18-Thr21, Gly27-Asp30, Asp32-Arg38, Gln45-Arg46, Gly50-Ala54, Lys56-Ser58, Gln65-Phe68 (Fig. 3a). The strands are arranged into an ‘amyloid key’ fold^22^ with three partially overlapping arches at residues Gly1-Thr21, Gly12-His36, and Gly50-Gly69 (Fig. 3b). The C-terminus of the fibril protein is disordered and truncated approximately at position 83^22^.

**Fig. 4:**
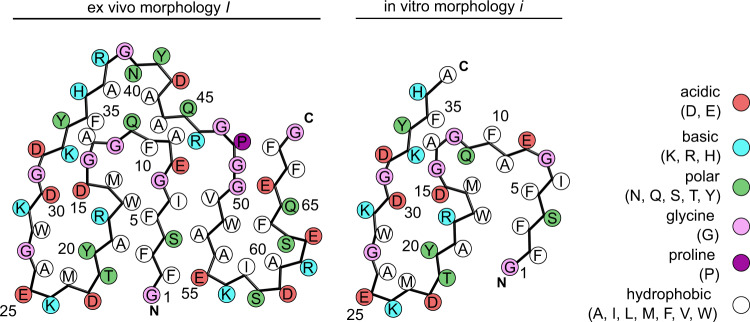
Schematic view of fibril protein packing in ex vivo fibril morphology I and in vitro fibril morphology i. Comparison of complementary fibril protein packing in ex vivo fibril morphology I and in in vitro fibril morphology i.

The ordered core of the in vitro fibrils is considerably smaller and formed by residues 1-37 (Fig. 3a). The C-terminal 66 residues of SAA1.1 are present in the fibril but conformationally disordered. The in vitro fibril contains seven β-strands (β1 to β7) at residues Phe2-Ile6, Glu8-Gln11, Asp15-Met16, Arg18-Thr21, Met23-Lys24, Ala26-Asp30, and Asp32-His36 (Fig. 3b). Residues 1–37 form two partially overlapping arches (Gly1-Thr21 and Gly12-His36), reminiscent of the ex vivo fibril (Fig. 3c). Residues Gly12-His36 present essentially identical conformations in the ex vivo and in vitro fibrils with buried ionic interactions at residues Asp15 and Lys33, as well as Arg18 and Asp30 and a small hydrophobic core formed by residues Tyr20, Met23, and Trp28 (Fig. 4). The N-terminal arch is notably different in the ex vivo and in vitro fibrils. Ex vivo fibrils contain residues Phe3 and Phe5 in a tightly packed arrangement with Ala19 and Thr21 (Fig. 3c). In vitro fibrils show interactions of residues Phe3 and Phe5 that are mainly formed with Trp17 (Fig. 3c).

The result of this revised packing is the formation of a large cavity within the center of the PF of the in vitro fibrils (Fig. 2c). Residues Arg38-Ala54 are no longer able to wrap around the N-terminal 37 residues and the interaction site between adjacent PFs in the ex vivo fibrils (Asp59-Arg61) is lost in the in vitro fibrils (Supplementary Fig. 3, Supplementary Fig. 6). Gly7 shows a different orientation in the two fibrils. Its Cα hydrogens are oriented towards the hydrophobic core of the ex vivo fibrils and towards the solvent in the in vitro fibrils (Fig. 3c).

### Ex vivo fibrils are more proteolytically resistant

**Fig. 5:**
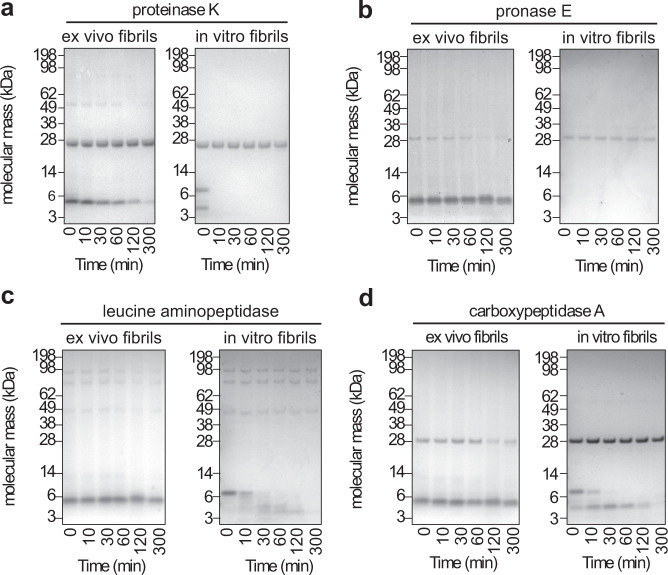
Stability of the fibrils against proteolysis. Coomassie-stained denaturing protein gels of ex vivo AA amyloid fibrils and in vitro formed SAA1.1 fibrils that were incubated with different proteases for up to 300 min: proteinase K (**a**, *n* = 3) and three other proteases (pronase E (**b**), leucine aminopeptidase (**c**) and carboxypeptidase A (**d**), *n* = 1). Bands above 14 kDa originate from the proteases. Due to the harsh proteolytic conditions used in this experiment there is discernible degradation of in vitro fibrils during sample workup for electrophoresis (0 min), specifically with proteinase K and pronase E.

Ex vivo fibrils differ not only in their structure from in vitro fibrils, they are also more protease resistant. Eight microgram proteinase K digest 4 μg in vitro fibrils within 10 min (Fig. 5a). Ex vivo fibrils, by contrast, resist these proteolytic conditions for more than 300 min (Fig. 5a). Consistent results were obtained in this regard with three other endoproteases or exoproteases: pronase E, leucine aminopeptidase and carboxypeptidase A (Fig. 5b–d). With all proteases we find ex vivo fibrils to be more resistant to proteolytic digestion than the tested in vitro fibrils from SAA1.1 protein. The different proteolytic stability does not correlate with major differences in the resistance to the chaotropic denaturant guanidine hydrochloride (GdnHCl). Both SAA1.1-derived fibril samples become fully denatured in 3 M GdnHCl (Supplementary Fig. 7a), and they are much less stable than a sample of light chain-derived fibril from human systemic AL amyloidosis. AL amyloid fibrils are not majorly disrupted even if the GdnHCl concentration is raised to 4 M (Supplementary Fig. 7b).

## Discussion

In this study we have analyzed the structure of amyloid fibrils that are derived from murine SAA1.1 protein. We report three cryo-EM structures of SAA1.1-derived amyloid fibrils: one that was extracted from the diseased murine tissue and two that were formed from murine SAA1.1 protein in vitro. The resolutions of the three structures range from 2.73 Å to 3.5 Å (Supplementary Table 2), indicating lack of information regarding structural details, such as bond angles and side chain conformations. Moreover, cryo-EM structures can generally suffer from beam damage artifacts^23^, reconstruction errors^24^ or the lack of density due to structural flexibility^25^. Despite these limitations, we could establish the polymorphic nature of analyzed fibrils and the structural differences between in vitro and ex vivo fibrils. While there may be areas of amyloid research in which exact 3D structures of ex vivo amyloid fibrils are not required, such information can be very important, for example, when analyzing the molecular basis of disease and the misfolding of body-own proteins into their pathogenic states.

Several recent studies report structural differences between fibrils formed inside a test tube and fibrils extracted from diseased tissue^4–8^. Morphological differences depend, on the one hand, on the primary structure of the aggregating polypeptide chain and on the other hand on the conditions under which fibril formation occurs. Murine AA fibril proteins show a heterogeneous C-terminal truncation occurring e.g., at residues 76, 82, and 83^22^, which suggests that the fibril morphology might be affected by these primary structural differences from full-length SAA1.1. However, a previous analysis of C-terminally truncated variants of murine SAA1.1 protein could not find any strong influence on the fibril morphology^26^ and was unable to obtain in vitro fibril morphologies that matched those extracted from diseased tissue. A possible reason is that the protein C-terminus does not participate in forming the fibril core (Fig. 3). The different morphologies of in vitro fibrils may thus have arisen from fibril formation conditions that did not exactly match the conditions of fibril formation in vivo.

There are pathological features of systemic AA amyloidosis that can only be explained by assuming that pathology depends on specific fibril morphologies. An example is the resistance of CE/J mice to developing AA amyloidosis. This resistance arises from the expression of the SAA2.2 protein variant^27^. SAA2.2 is incompatible with the protein fold of the ex vivo fibril as the Gly7His mutational change of the variant protein disrupts the hydrophobic core of the pathogenic fibril^22^. Surprisingly, however, SAA2.2 can readily form fibrils in vitro^28^, demonstrating that the Gly7His mutation does not simply block fibril formation but that it is selectively unfavorable to the pathogenic fibrils. Our current structures support this conclusion by showing that Gly7 is, in contrast to the ex vivo fibrils, solvent exposed. That is, a Gly7His replacement could be tolerated by this fibril structure but this fibril morphology is not relevant for disease.

If disease depends on the formation of specific fibril morphologies, what might determine their pathogenicity? In vitro fibril proteins encompass residues 1–37 within a stable conformation, whereas ex vivo fibril proteins show stable conformation at residues 1–69 (Fig. 3a). Similarly, ex vivo fibril proteins show a higher β-sheet content of 41 residues compared with 27 residues in the in vitro fibrils (Fig. 3a). These differences might be assumed to result in a higher thermodynamic stability of pathogenic fibrils, which would be in accord with a recent study demonstrating that ex vivo fibrils from transthyretin protein are more resistant to guanidine denaturation (and more thermodynamically stable) than the in vitro fibrils from this protein^29^.

However, in vitro and ex vivo SAA1.1-derived fibrils are relatively unstable in guanidine solution and become fully denatured in 3 M GdnHCl. There is also no major difference in the GdnHCl stability of ex vivo and in vitro fibrils from SAA1.1 protein. The two SAA-derived fibrils are much less stable than a sample of light chain-derived amyloid fibrils from a patient with systemic AL amyloidosis that resist denaturation in up to 4 M GdnHCl (Supplementary Fig. 7). In the case of the ex vivo fibrils from transthyretin it was found that even guanidine isothiocyanate had to be used to fully denature these assemblies^29^. We conclude that properties other than simple thermodynamic stability must be more relevant when rationalizing the pathological involvement of the observed ex vivo amyloid fibrils.

One possibility is a higher kinetic stability of ex vivo fibrils. Another possibility is proteolysis. Amyloid fibrils were previously found to be more proteolytically resistant than the natively folded precursor protein^30^ and there is evidence that cellular proteolysis is an important trigger for development of amyloidosis^31^. Using four different endoproteases and exoproteases we here show that ex vivo fibrils are considerably more stable than the tested in vitro fibrils (Fig. 5a–d). These observations correspond to data obtained with Aβ amyloid fibrils, which were found to be more protease-resistant when purified from patient tissue than when formed in vitro^6,32^. In addition, there is also long-standing evidence that proteolytic resistance is a feature of amyloid deposits in systemic amyloidosis, although no direct comparison was presented in these studies on in vitro and ex vivo fibrils from the same precursor protein^33,34^. Ultimately, proteolytic resistance is also one of the biochemical hallmarks of infectious prions^35^.

**Fig. 6:**
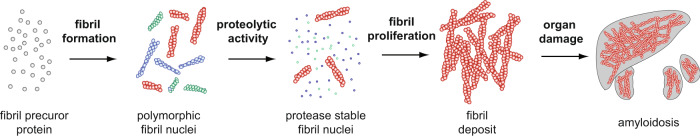
Schematic representation of the proteolytic selection mechanism. Proliferation and subsequent organ deposition of proteolytically selected pathogenic fibril morphologies. The protease unstable morphologies have been depicted in blue and green while the protease stable morphology is shown in red.

Based on these observations we suggest that the following mechanism may underlie the formation of pathogenically relevant amyloid fibril morphologies (Fig. 6): a fibril precursor protein such as SAA1.1 is able to adopt a range of different amyloid fibril structures and fibril seeds. These variations depend on stochastic fluctuations, kinetic factors, as well as the precise chemical and physical conditions under which fibril formation takes place, including the action of molecular cofactors. The morphologies differ in a number of structural properties, including their proteolytic resistance. Fibrils with a higher proteolytic resistance are more likely to survive for longer periods of time within a living tissue or organ. They may have an increased possibility to proliferate in a prion-like fashion such that a specific fibril morphology can predominate and cause pathology to the surrounding tissue. In other words, pathogenic amyloid fibril morphologies may have been selected inside the body by their ability to escape endogenous clearance mechanisms. We here term this mechanism proteolytic selection.

Amyloid fibrils and their deposits are key players in the development of systemic amyloid diseases and account for important parts of the observed pathology. However, additional factors may contribute, such as the non-fibril components of amyloid deposits or oligomeric fibril precursors with cell-toxic effects. While more work is required to test the aforementioned proteolytic selection mechanism in systemic AA amyloidosis and other protein misfolding diseases, our study generally supports the view that systemic AA amyloidosis is a conformational disease and that specific fibril morphologies are responsible for its pathology. The ramification of this conclusion is that targeting these specific fibril architectures with appropriate inhibitors may represent a plausible strategy to combat protein misfolding diseases. For several types of systemic amyloidosis high resolution structural information is now available for the underlying pathogenic agents^11,22,36,37^.

## Methods

### Source of recombinant murine SAA1.1 protein

Murine SAA1.1 protein was recombinantly expressed in *Escherichia coli* RV308 as described previously^38^. In brief, the coding region of SAA1.1 was cloned to the C-terminus of a His-tagged maltose-binding protein in a pMAL-c2X vector (New England Biolabs) separated by a cleavage site for tobacco etch virus protease. Protein purification was done in five steps: (i) amylose resin high flow using a linear gradient of 0–10 mM maltose in Tris buffer A (20 mM Tris/HCl, pH 7.5, 200 ml NaCl) (New England Biolabs), (ii) nickel-sepharose fast flow (GE Healthcare) chromatography using a linear gradient of 0–250 mM imidazol in Tris buffer B (20 mM Tris/HCl, pH 8.0, 150 mM NaCl), (iii) fusion protein cleavage by overnight incubation with tobacco etch virus protease at 34 °C in Tris buffer B (iv) nickel chelate chromatography (same conditions as ii) to separate SAA1.1 from the fusion protein and maltose-binding protein, and (v) Source 15 RPC (GE Healthcare) reversed-phase chromatography using 0–86% (v/v) linear gradient of acetonitrile in 0.1% (v/v) trifluroacetate. The purified protein was lyophilized using an alpha 2-4 LD plus freeze dryer (Christ).

### Formation of in vitro fibrils

Recombinant SAA1.1 protein was incubated at 0.2 mg/ml concentration in 10 mM Tris(hydroxymethyl)aminomethane (Tris) buffer pH 8.5 for 48 h at 37 °C. The incubation was carried out in a black 96-well plate (Greiner Bio-One) at 37 °C in a FLUOstar OMEGA plate reader (BMG Labtech). The sample volume in each well was 100 µl, and the plate was agitated every 30 min by double orbital shaking for 10 s at 100 rpm.

### Extraction of amyloid fibrils from diseased tissue

The AL fibril extraction was performed from the heart muscle tissue of a patient (FOR007) suffering from λ-AL amyloidosis (heart explanted at an age of 50 years). The AA fibrils were extracted from the liver of a mouse suffering from systemic AA amyloidosis. Two hundred and fifty miligram of fat free tissues was diced and washed five times with 0.5 ml Tris calcium buffer (20 mM Tris, 138 mM NaCl, 2 mM CaCl_2_, 0.1% NaN_3,_ pH 8.0). The solution was centrifuged for 5 min at 3100 × *g* and 4 °C after each wash. The pellet was resuspended in 1 ml of freshly prepared 5 mg/ml solution of *Clostridium histolyticum* collagenase (Sigma) in Tris calcium buffer (with 1 tablet of ethylenediaminetetraacetic acid (EDTA)-free protease inhibitor in 7 ml Tris calcium buffer). After an overnight incubation in horizontal position, at 37 °C with shaking at 700 rpm, the sample was centrifuged for 30 min at 3100 × *g* and 4 °C. The pellet was resuspended in 0.5 ml buffer containing 20 mM Tris, 140 mM NaCl, 10 mM EDTA, 0.01% NaN_3_. This solution was again centrifuged for 5 min at 3100 × *g* and 4 °C and the process was repeated until the supernatant was clear. The resulting pellet was resuspended several times in cold Millipore water and then centrifuged for 5 min at 3100 × *g* and 4 °C. The fibrils in the supernatant were used for the GdnHCl denaturation study. The mice used in the study had the following housing conditions: humidity: 57%, temperature: 22.4 °C, dark/light cycle: 12 h dark/12 h light. The study regarding human tissue was approved by the ethical committee of Ulm University (203/18) and informed consent was obtained from the participant. We have complied with all relevant ethical regulations for all animal experiments and these were approved by the Regierungspräsidium Tübingen.

### Platinum shadowing

Formvar and carbon coated 200 mesh copper grids (Plano) were glow-discharged using a PELCO easiGlow glow discharge cleaning system (TED PELLA). A 5 μl droplet of the in vitro fibrils (0.2 mg/ml) was placed onto a grid and incubated for 30 s at room temperature. Excessive solution was removed with filter paper (Whatman). Grids were washed three times with water and dried at room temperature for 30 min. Platinum was evaporated at an angle of 30° using a Balzers TKR 010 to form a 1 nm thick layer on the sample. Grids were examined in a JEM-1400 transmission electron microscope (JEOL), operated at 120 kV.

### Cryo-EM

The reconstruction of murine AA amyloid fibril morphology II was obtained by re-analyzing a previously recorded cryo-EM data set that was used to obtain fibril morphology I^22^. For the in vitro fibril cryo-EM data set, a 3.5 µl aliquot (0.2 mg/ml) was applied to glow-discharged holey carbon coated grids (400 mesh C-flat 2/1), blotted with filter paper and plunge-frozen in liquid ethane using a Vitrobot Mark 3 (Thermo Fisher Scientific). Grids were screened using a JEM-2100 transmission electron microscope (JEOL) at 200 kV. Images were acquired using a K2-Summit detector (Gatan) in counting mode on a Titan Krios transmission electron microscope (Thermo Fisher Scientific) at 300 kV. Data acquisition parameters are listed in Supplementary Table 1.

### Helical reconstruction

Movie frames were corrected for gain reference using IMOD^39^. Motion correction and dose-weighting was done using MOTIONCOR2^40^. The contrast transfer function was estimated from the motion-corrected images using Gctf^41^. Helical reconstruction was performed using RELION 2.1^24^ (in vitro fibril morphology i and ex vivo fibril morphology II) or RELION 3.0 (in vitro fibril morphology ii)^42^. Fibrils were picked manually in a morphology-specific fashion. Segments were extracted according to Supplementary Table 2. Reference-free 2D classification was used to select class averages showing the helical repeat along the fibril axis. The initial 3D models for in vitro fibril morphology i and ex vivo fibril morphology II were generated de novo from a small subset (200 particles per class) of the selected class averages using the Stochastic Gradient Descent algorithm implementation in RELION. Both initial models were low-pass filtered to 20 Å and used as reference in the 3D auto-refinement. The initial 3D model for in vitro fibril morphology ii was generated by low-pass filtering the final density of in vitro fibril morphology i to 30 Å. Primary fibril models were created using 3D auto-refinement. The resulting reconstructions showed clearly separated β-sheets (*x*–*y* plane) and partially resolved β-strands along the fibril axis. The generated primary models indicated a pseudo-2_1_ screw symmetry for in vitro fibril morphology i, C2 symmetry for in vitro fibril morphology ii and C1 symmetry for ex vivo fibril morphology II. Imposing these symmetries during reconstruction yielded clearly separated β-strands and side-chain densities. 3D classification with local optimization of helical twist and rise was used to further select particles in the in vitro fibril data set for a final high-resolution auto-refinement. The best 3D classes were selected manually and reconstructed with local optimization of helical parameters using 3D auto-refinement. Due to the low number of segments, the 3D classification step was omitted for the ex vivo fibril morphology II. All 3D classification and auto-refine processes were carried out using a central part of 10% of the intermediate asymmetrical reconstruction^24^. The final reconstructions were post-processed with a soft-edge mask and *B*-factor sharpened. The resolutions of the individual reconstructions were estimated from the FSC at 0.143 between two independently refined half-maps.

### Model building, refinement and evaluation

The 3D maps of in vitro and ex vivo fibril morphologies were sharpened with model based and model free automated sharpening procedures, respectively, using the phenix.auto_sharpen tool implemented in Phenix^43^. The model for in vitro fibril morphology i was built de novo using the program Coot^44^. A poly-L-Ala chain was traced along the main chain density. The side chains were mutated according to residues 1–37 of the SAA1.1 protein sequence. Once a satisfactory main chain and reasonable side-chain density fit was achieved for one polypeptide chain, a fibril stack comprising twelve polypeptide chains was assembled using UCSF Chimera^45^. Hydrogen atoms were added to the structure using the phenix ready-set program^43^. The structure was refined manually with Coot and using phenix.real_space_refine with non-crystallographic symmetry (NCS) constraints, Ramachandran, atomic displacement parameter and rotamer restraints. Secondary structure restraints were implied using manually defined beta sheets. The validation output generated using MolProbity^46^ and comprehensive validation tool in Phenix^43^ were analyzed for atomic clashes, rotamer and Ramachandran outliers and model geometry. This process of iterative refinement and modeling was repeated until the refinement converged to produce reasonable density to model fit. In vitro morphology ii and ex vivo morphology II were modeled as described above using in vitro morphology i and ex vivo morphology I^22^ as the starting models although the automated refinement was performed in the absence of NCS constraints. The structural statistics for refinement and model building are listed in Supplementary Table 3.

### Image representation

Image representations of reconstructed densities and refined models were created with UCSF Chimera^45^ and PyMOL^47^.

### Quantification of fibril morphologies

Fibrils were morphologically classified according to their widths and cross-over structures as measured with the program Fiji^48^. The relative frequency of each fibril morphology was determined by analyzing and counting all visible fibrils within 100 randomly chosen cryo-EM images, whereby only fibrils longer than 150 nm were counted as such.

### Fibril denaturation with GdnHCl

Twenty microgram of ex vivo murine AA or human AL fibrils (in water) or in vitro fibrils formed from recombinantly expressed murine SAA1.1 protein were transferred in ultracentrifuge tubes and centrifuged for 1 h at 100,000×*g* and 4 °C. The supernatants were discarded, and the pellets were resuspended in 30 μl GdnHCl solution containing 100 mM Tris buffer (pH 7). The GdnHCl concentrations used in this experiment varied as indicated in Supplementary Fig. 7. The tubes were sealed with Parafilm and the samples were incubated for 3 h at room temperature before they were centrifuged once more for 1 h at 100,000 × *g* and 20 °C. The supernatants were discarded, and the pellets were resuspended in 30 μl Millipore water for analysis with denaturing gel electrophoresis.

### Proteolytic digest of fibrils

For proteinase K treatment, a 180 µl aliquot from a stock solution of ex vivo or in vitro fibrils at 0.2 mg/ml concentration was mixed with 20 µl 200 mM Tris buffer, pH 8.0, containing 1.4 M NaCl and 20 mM CaCl_2_. 0.4 µl proteinase K (20 mg/ml, Fermentas) was added to the mixture and incubated at 37 °C. Samples of 20 µl were taken after 0, 10, 30, 60, 120, and 300 min and the proteinase K activity was stopped by adding 0.5 µl of 200 mM phenylmethane sulfonyl fluoride (Roth) prepared in methanol (Roth) to the reaction mixture and incubation for 10 min at room temperature. For pronase E, leucine aminopeptidase and carboxypeptidase A treatment, a 180 µl aliquot from a stock solution of ex vivo or in vitro fibrils at 0.2 mg/ml concentration was mixed with 10 µl 2 M Tris buffer, pH 7.5. Ten microliter of the respective protease (800 µg/ml) was added to the mixture and incubated at 37 °C. Samples of 20 µl were taken after 0, 10, 30, 60, 120, and 300 min and the protease activity was stopped by adding 3 µl of a protease inhibitor cocktail [one protease inhibitor EDTA-free tablet (cOmplete^TM^, Roche) in 7 ml distilled water] to the reaction mixture and incubation for 10 min at room temperature. The proteolytic digestion products were analyzed using denaturing protein gel electrophoresis.

### Denaturing protein gel electrophoresis

Nine microliter of samples to be analyzed were mixed with 3 μl of 4× NuPAGE LDS Sample Buffer (Thermo Fischer Scientific) via vortexing. The samples were incubated at 95 °C for 10–15 min in a heating block. Ten microliter of samples and 5 to 8 μl of BlueEasy prestained protein marker were loaded into each well of a 4–12% NuPAGE Bis-Tris gel (Thermo Fischer Scientific). Electrophoresis was performed at 180 V for 35 min at room temperature. The gels were stained for 1 h at room temperature with Coomassie staining solution comprising 2.5% (w/v) Coomassie Brilliant Blue R250, 30% (w/v) Ethanol and 10% (v/v) acetic acid and destained using 20% (v/v) ethanol, 10% (v/v) acetic acid solution (source data are provided as Source Data file).

### Reporting summary

Further information on research design is available in the Nature Research Reporting Summary linked to this article.

## Supplementary information

Supplementary Information

Reporting Summary

## Data Availability

The reconstructed cryo-EM maps were deposited in the Electron Microscopy Data Bank (EMDB) with the accession codes EMD-11162 (in vitro morphology i), EMD-11163 (in vitro morphology ii) and EMD-11164 (ex vivo morphology II). The coordinates of the fitted atomic models were deposited in the Protein Data Bank (PDB) under the accession codes 6ZCF (in vitro morphology i), 6ZCG (in vitro morphology ii) and 6ZCH (ex vivo morphology II) (ref. ^49^). The following previously published coordinates were used in Fig. 1: PDB 6DSO^50^. The data that support the findings of this study are available from the corresponding author upon reasonable request. The source data associated with following figures are provided with this paper: Fig. 5, Supplementary Figs. 1, 4, and 7. The unique materials used in this study are available from the corresponding author on reasonable request. Source data are provided with this paper.

## Source data

Source Data
