# Supplementary material for: AA amyloid fibrils from diseased tissue are structurally different from in vitro formed SAA fibrils
Source: Nat Commun. 2021 Feb 12;12:1013. doi: 10.1038/s41467-021-21129-z (PMC7881110; doi:10.1038/s41467-021-21129-z)
Supplement: Supplementary file 1 — Supplementary Information [file 41467_2021_21129_MOESM1_ESM.pdf]

Supplementary information

AA amyloid fibrils from diseased tissue are structurally different from in vitro formed SAA fibrils

A. Bansal, M. Schmidt et al.

Supplementary Figure 1

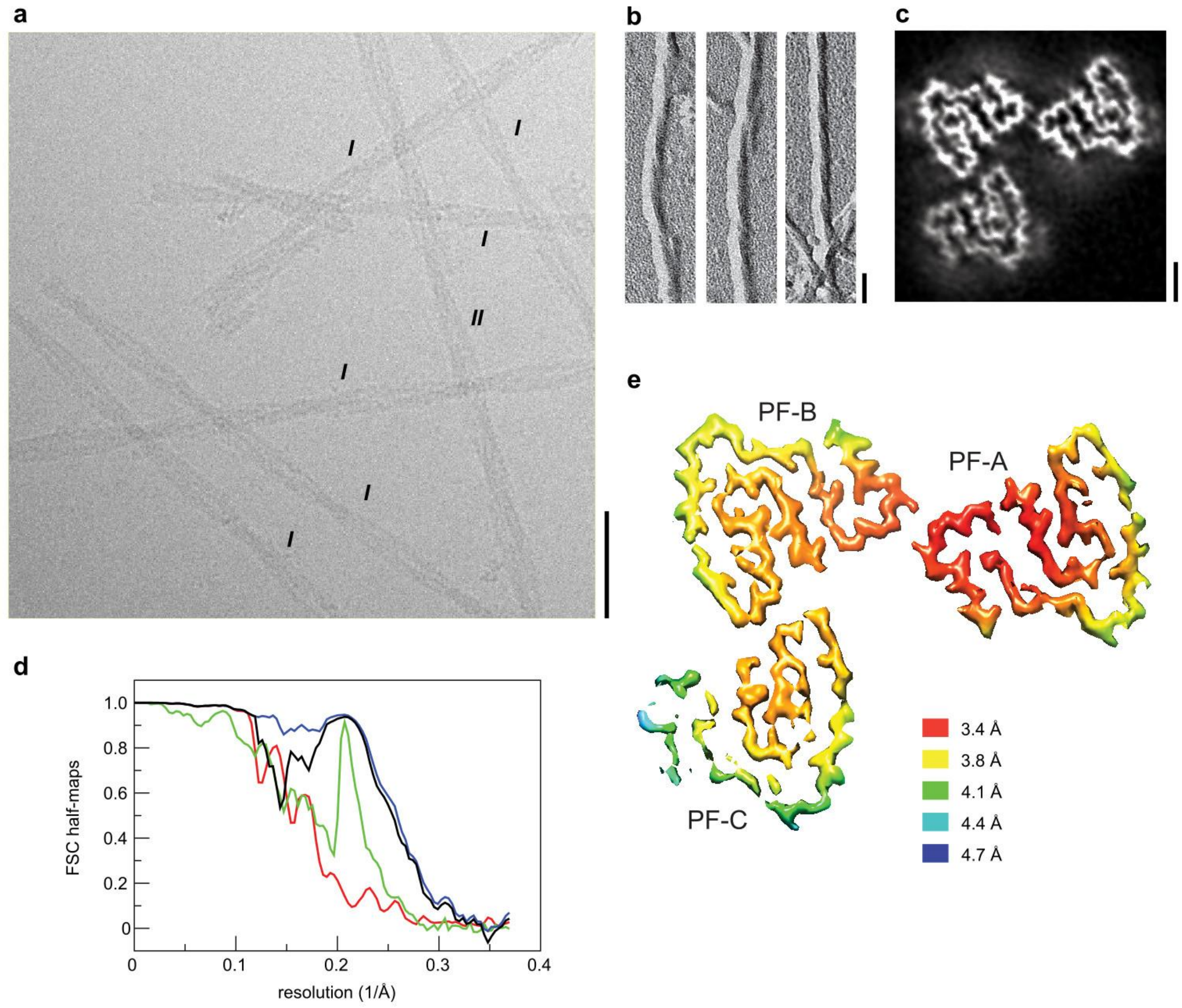

Supplementary Fig. 1.

Cryo-EM structure of ex vivo fibril morphology *II*.

(a) Cryo-EM image showing fibril morphologies *I* and *II*. Scale bar: 50 nm. 100 cryo-EM images were used for the morphological analysis. (b) TEM images of platinum side shadowed fibrils of morphology *II*. Scale bar: 50 nm. Three platinum shadowed images were analysed for determining the handedness. (c) A 6.75 Å cross sectional slice of the 3D map. Scale bar: 20 Å. 15,505 segments were used for 3D reconstruction. (d) FSC between the two half-maps. Black, FSC corrected; green, FSC unmasked maps, blue, FSC masked maps; red,

corrected FSC phase randomized masked maps. (e) Local resolution map of the fibril cross section.

Supplementary Figure 2

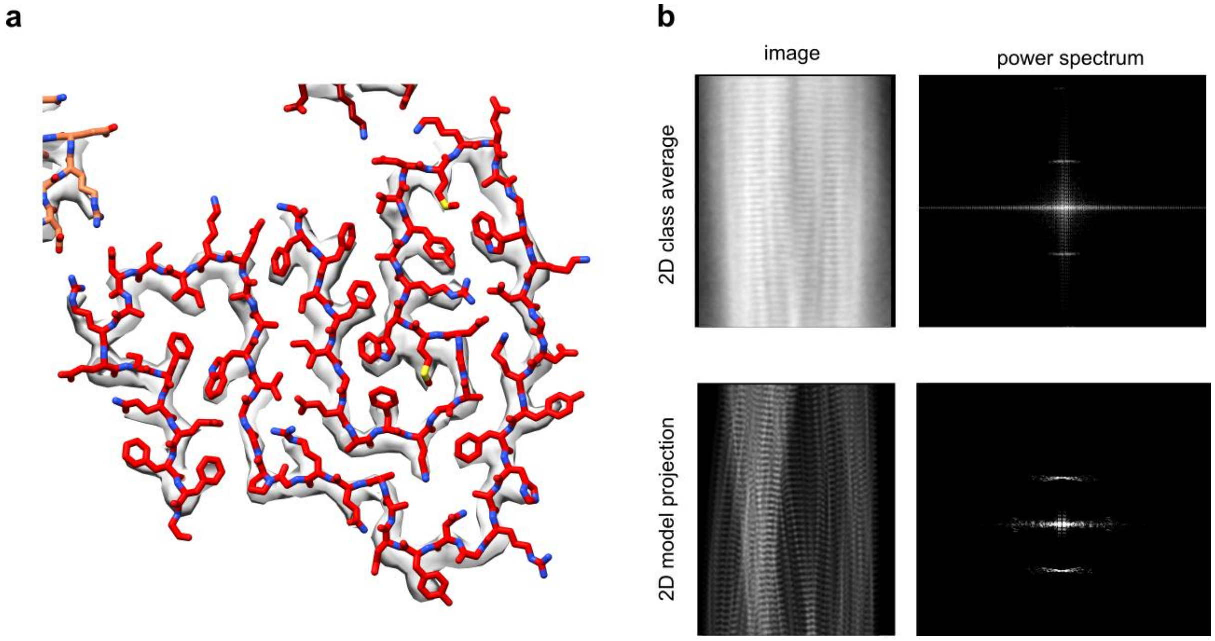

Supplementary Fig. 2.

Comparison of the model of morphology *II* with the 3D map and 2D class averages.

(a) Fit of model (red) into 3D map (grey). (b) Comparison of a 2D class average and a 2D projection of corresponding segments of the model along with their power spectra (right side).

Supplementary Figure 3

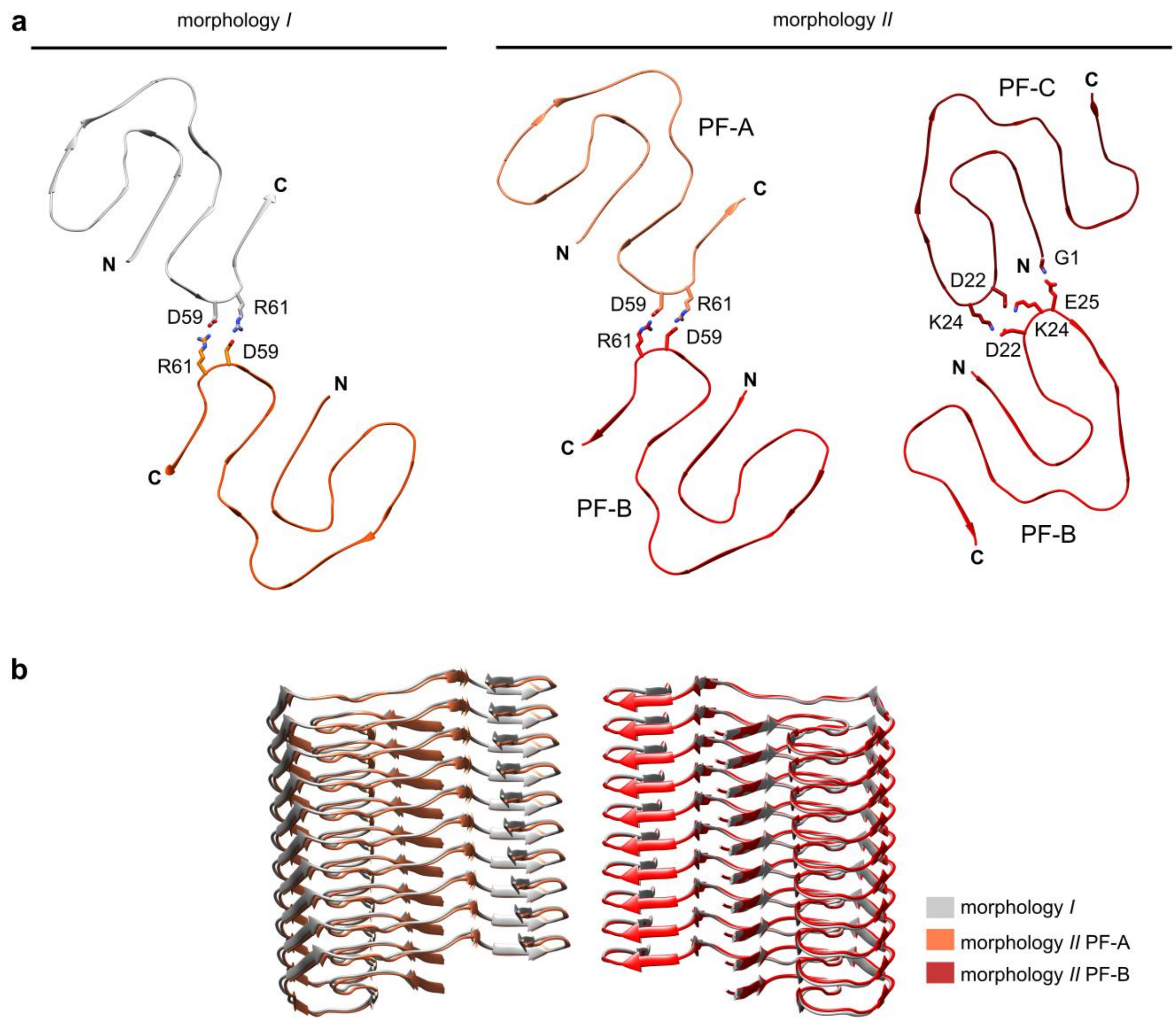

Supplementary Fig. 3.

Contact sites between the PFs in the ex vivo fibril morphologies *I* and *II*.

(a) Relative arrangement of the PFs in the ex vivo fibril morphologies *I* and *II* as indicated in the figure. Residues at the contact site are shown in sticks. (b) Overlay of 10 fibril layers of morphology *I* and *II*. The PFs of morphology *I* are colored orange and grey in the two panels, while morphology *II* is colored coral (PF-A), red (PF-B) and dark red (PF-C).

Supplementary Figure 4

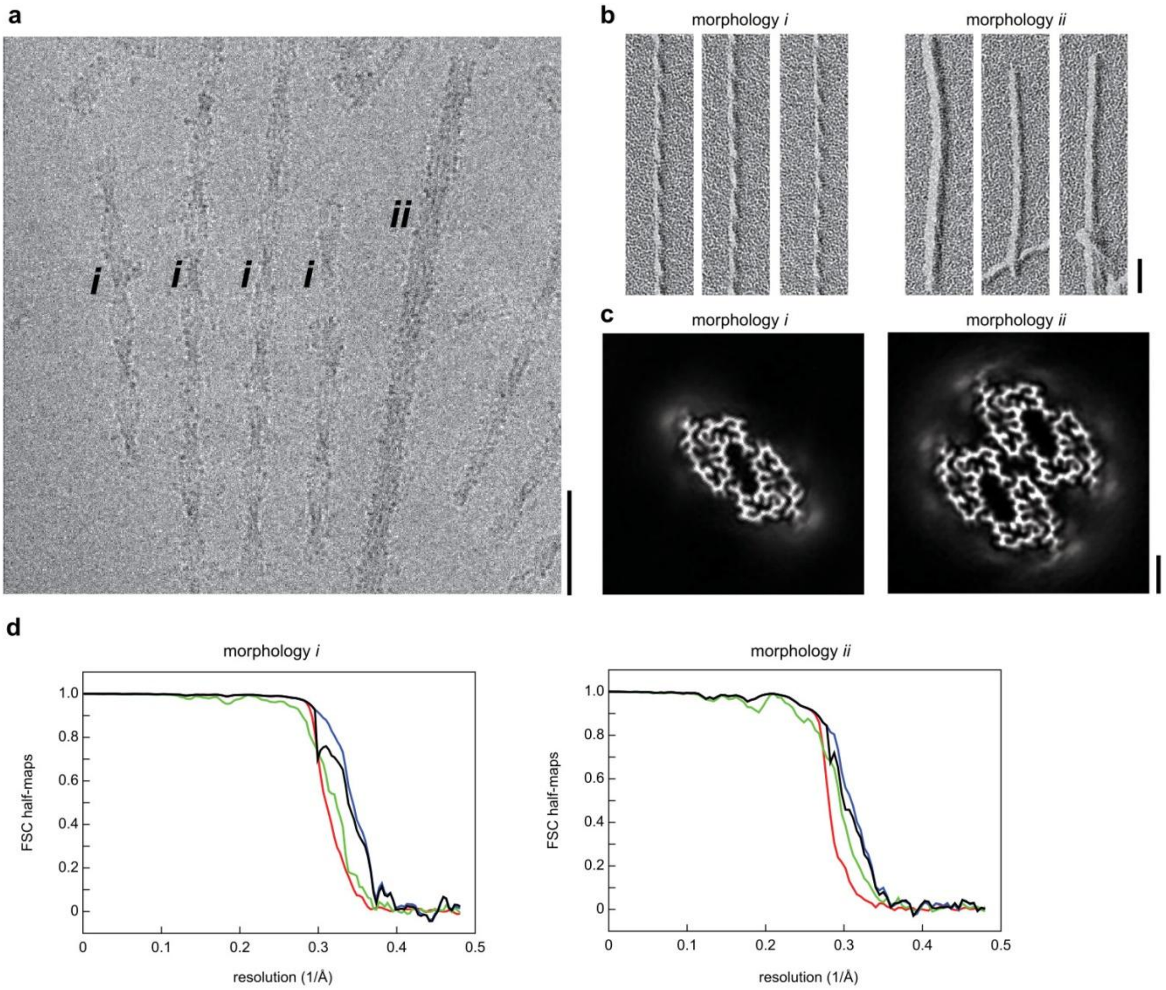

Supplementary Fig. 4.

Cryo-EM structure of the in vitro fibril morphologies *i* and *ii*.

(a) Cryo-EM image of in vitro fibrils showing two fibril morphologies. Scale bar: 50 nm. 100 EM images were used for morphological analysis. (b) TEM images of the fibrils after platinum side shadowing. Scale bar: 50 nm. Three platinum shadowed images were analysed for determining handedness. (c) 6.24 Å cross-sectional slices of the 3D maps. Scale bar: 20 Å. 93,347 and 21,355 segments were used for final 3D reconstruction of morphology *i* and morphology *ii*, respectively. (d) FSC between the two half-maps. Black, FSC corrected;

green, FSC unmasked maps, blue, FSC masked maps; red, corrected FSC phase randomized masked maps.

Supplementary Figure 5

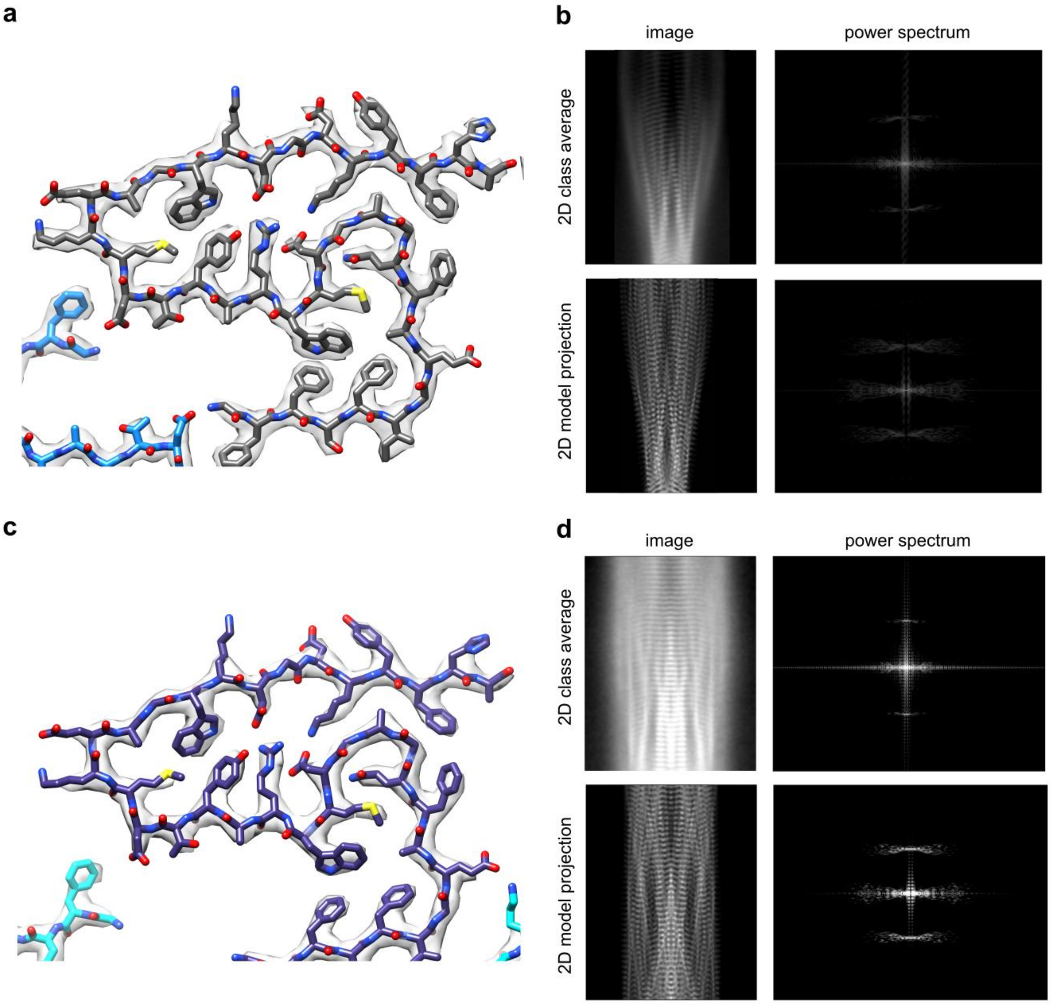

Supplementary Fig. 5.

Comparison of the models of the in vitro fibrils with the 3D maps and 2D class averages. (a) Fit of the model of the in vitro fibril morphology *i* (dim grey) into the 3D map (grey). (b) Comparison of a 2D average and a 2D projection of the in vitro fibril morphology *i*. The left column shows the real space images, the right column shows the respective power spectra. (c) Fit of the model of the in vitro fibril morphology *ii* (dark slate blue) into the 3D map (grey). (d) Comparison of a 2D average and a 2D projection of the in vitro fibril morphology *ii*. The left column shows the real space images, the right column shows the respective power spectra.

Supplementary Figure 6

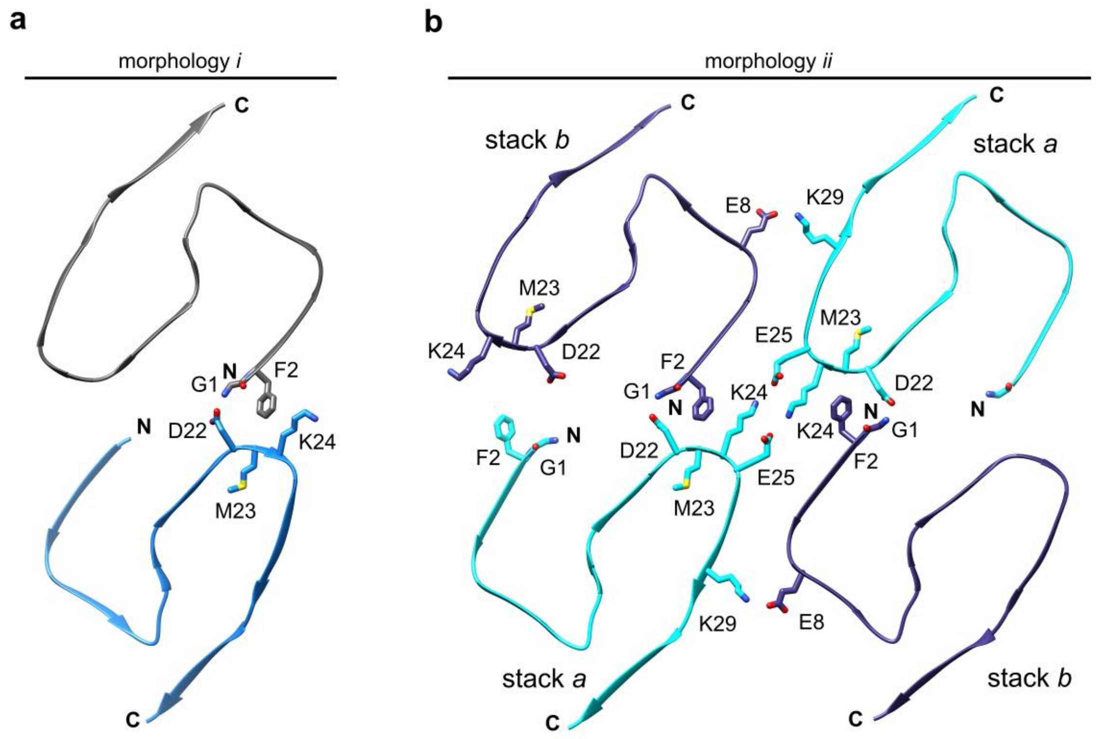

Supplementary Fig. 6.

Contact sites between the stacks of the in vitro fibril morphologies *i* and *ii*.

Relative arrangement of the protein stacks in the in vitro fibril morphologies. The residues at the contact site are shown as sticks for (a) morphology *i* (with the two PFs colored dodger blue and dim grey) and (b) morphology *ii* (with PFs of stacks *a* and stacks *b* being colored cyan and dark slate blue, respectively).

Supplementary Figure 7

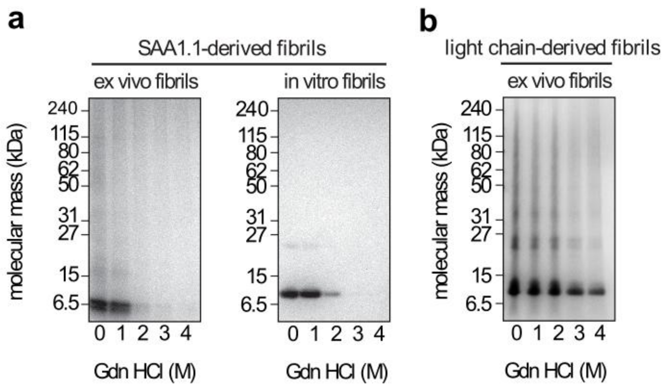

Supplementary Fig. 7.

Stability of fibrils against guanidine denaturation.

Coommassie stained denaturing protein gels with the pellet fraction of ex vivo or in vitro SAA1.1-derived fibrils (a) or light chain-derived amyloid fibrils from systemic AL amyloidosis (b) after their incubation in GdnHCl-containing solutions at different concentrations as indicated. The experiment was carried out thrice for in vitro and once for ex vivo SAA1.1 derived fibrils and twice for light chain derived amyloid fibrils.

Supplementary Tables

Supplementary Table 1

Microscope	Titan Krios (Thermo Fisher Scientific)
Camera	K2 Summit (Gatan)
Acceleration voltage (kV)	300
Magnification	x 130,000
Defocus range (µm)	-0.5 to -2.5
Dose rate (e^-/Å^2/s)	3.33
Number of movie frames	40
Exposure time (s)	12
Total electron dose (e^-/Å^2)	40
Pixel size (Å)	1.04

Supplementary Table 1.

Structural statistics of cryo-EM data collection of the in vitro fibrils.

Supplementary Table 2

	Ex vivo fibril morphology *II*	In vitro fibril morphology *i*	In vitro fibril morphology *ii*
Box size (pixel)	210	270	200
Inter box distance (Å)	28	28	29
Number of extracted segments	15,530	94,220	80,724
Number of segments after 2D classification	15,505	94,220	80,694
Number of segments after 3D classification	15,505	93,347	21,355
Resolution, 0.143 FSC criterion (Å)	3.5	2.73	2.95
Map sharpening B-Factor (Å^2)	-53	-86	-97
Symmetry	C_1	C_1	C_2
Helical rise (Å)	4.81	2.37	4.75
Helical twist (°)	-1.09	178.93	-1.6

Supplementary Table 2.

Structural statistics of image processing of the ex vivo and in vitro fibril morphologies.

Supplementary Table 3

	Ex vivo fibril morphology *II*	In vitro fibril morphology *i*	In vitro fibril morphology *ii*
Initial model used	6DSO.PDB	De novo	6ZCF.PDB
Model resolution (Å)<br>FSC threshold at 0.143	3.46	2.68	2.92
Map sharpening *B* factor ($Å^2$)	25.16	19.3[a]	34.7[a]
Model composition<br>Non-hydrogen atoms<br>Protein residues<br>Ligands	 9882<br>1242<br>0	 3600<br>444<br>0	 7200<br>888<br>0
Mean *B* factor ($Å^2$)	80.93	17.17	37.76
R.m.s deviations<br>Bond length (Å)<br>Bond angle (°)	 0.007<br>0.874	 0.006<br>0.864	 0.007<br>0.828
Validation<br>Molprobity score<br>Clash score<br>Poor rotamers (%)	 2.14<br>11.85<br>0	 1.40<br>4.73<br>0	 1.73<br>5.47<br>0
Ramachandran plot<br>Favoured (%)<br>Allowed (%)	 90.05<br>9.95	 97.14<br>2.86	 93.33<br>6.67

Disallowed (%)	0	0	0
EMRinger score			
z score	12.00	9.54	15.26
score	4.31	5.62	6.36
Map CC			
CC_{mask}	0.82	0.87	0.87

Supplementary Table 3.

Structural statistics of model building and refinement.

[a] Overall map *B* value for model-based sharpened map.
