# Supplementary material for: AA amyloid fibrils from diseased tissue are structurally different from in vitro formed SAA fibrils
Source: Nat Commun. 2021 Feb 12;12:1013. doi: 10.1038/s41467-021-21129-z (PMC7881110; doi:10.1038/s41467-021-21129-z)
Supplement: Supplementary file 2 — Reporting Summary [file 41467_2021_21129_MOESM2_ESM.pdf]

nature research

Corresponding author(s): Marcus faendrich

Last updated by author(s): Dec 22, 2020

Reporting Summary

Nature Research wishes to improve the reproducibility of the work that we publish. This form provides structure for consistency and transparency in reporting. For further information on Nature Research policies, see our Editorial Policies and the Editorial Policy Checklist.

Statistics

For all statistical analyses, confirm that the following items are present in the figure legend, table legend, main text, or Methods section.

n/a	Confirmed	
☐	☒	The exact sample size (*n*) for each experimental group/condition, given as a discrete number and unit of measurement
☐	☒	A statement on whether measurements were taken from distinct samples or whether the same sample was measured repeatedly
☒	☐	The statistical test(s) used AND whether they are one- or two-sided<br>*Only common tests should be described solely by name; describe more complex techniques in the Methods section.*
☒	☐	A description of all covariates tested
☒	☐	A description of any assumptions or corrections, such as tests of normality and adjustment for multiple comparisons
☒	☐	A full description of the statistical parameters including central tendency (e.g. means) or other basic estimates (e.g. regression coefficient) AND variation (e.g. standard deviation) or associated estimates of uncertainty (e.g. confidence intervals)
☒	☐	For null hypothesis testing, the test statistic (e.g. *F*, *t*, *r*) with confidence intervals, effect sizes, degrees of freedom and *P* value noted<br>*Give P values as exact values whenever suitable.*
☒	☐	For Bayesian analysis, information on the choice of priors and Markov chain Monte Carlo settings
☒	☐	For hierarchical and complex designs, identification of the appropriate level for tests and full reporting of outcomes
☒	☐	Estimates of effect sizes (e.g. Cohen's *d*, Pearson's *r*), indicating how they were calculated

Our web collection on statistics for biologists contains articles on many of the points above.

Software and code

Policy information about availability of computer code

Data collection	SerialEM v3.5
Data analysis	RELION v2.1 and v3.0, MotionCor2 v1, Gctf v1.06, IMOD v.4.9.4 and v.4.9.12, COOT v0.8.9, PHENIX v1.16-3549 and v1.14, UCSF Chimera v1.13.1, PyMol v2.3.4, Fiji v2.0

For manuscripts utilizing custom algorithms or software that are central to the research but not yet described in published literature, software must be made available to editors and reviewers. We strongly encourage code deposition in a community repository (e.g. GitHub). See the Nature Research guidelines for submitting code & software for further information.

Data

Policy information about availability of data

All manuscripts must include a data availability statement. This statement should provide the following information, where applicable:

- Accession codes, unique identifiers, or web links for publicly available datasets
- A list of figures that have associated raw data
- A description of any restrictions on data availability

TThe reconstructed cryo-EM maps were deposited in the Electron Microscopy Data Bank (EMDB) with the accession codes EMD-11162 (in vitro morphology i), EMD-11163 (in vitro morphology ii) and EMD-11164 (ex vivo morphology II) The coordinates of the fitted atomic models were deposited in the Protein Data Bank (PDB) under the accession codes 6ZCF (in vitro morphology i), 6ZCG (in vitro morphology ii) and 6ZCH (ex vivo morphology II). The data that support the findings of this study are available from the corresponding author upon reasonable request.

Field-specific reporting

Please select the one below that is the best fit for your research. If you are not sure, read the appropriate sections before making your selection.

- [x] Life sciences
- [ ] Behavioural & social sciences
- [ ] Ecological, evolutionary & environmental sciences

For a reference copy of the document with all sections, see nature.com/documents/nr-reporting-summary-flat.pdf

Life sciences study design

All studies must disclose on these points even when the disclosure is negative.

Sample size	Murine: AA amyloid fibrils were extracted from NMRI mice. Human: AL amyloid fibrils were extracted from a heart tissue sample of a single patient. One sample in each case was sufficient to extract fibrils requisite for analyses.
Data exclusions	15,530 segments were extracted from the raw data and 5,505 segments were used for final reconstruction of ex vivo morphology II
Replication	Electron microscopy data for AA amyloid fibrils is based on a single mouse. 15,505 segments were used for final reconstruction of ex vivo morphology II. Guanidine hydrochloride denaturation study for AA and AL amyloid fibrils is based on a single mouse and single human patient. The experiment was carried out thrice for in vitro and once for ex vivo SAA1.1 derived fibrils and twice for light chain derived amyloid fibrils. Experiment for analysis of fibril stability against proteolysis was carried out thrice using proteinase K and once using other proteases. All the attempts at replication were successful. The experiments carried out once were due to limited amount of available material.
Randomization	Not relevant to study. Single case Study.
Blinding	Not relevant to study. Single case Study.

Reporting for specific materials, systems and methods

We require information from authors about some types of materials, experimental systems and methods used in many studies. Here, indicate whether each material, system or method listed is relevant to your study. If you are not sure if a list item applies to your research, read the appropriate section before selecting a response.

Materials & experimental systems

n/a	Involved in the study	
x		Antibodies
x		Eukaryotic cell lines
x		Palaeontology and archaeology
	x	Animals and other organisms
	x	Human research participants
x		Clinical data
x		Dual use research of concern

Methods

n/a	Involved in the study	
x		ChIP-seq
x		Flow cytometry
x		MRI-based neuroimaging

Animals and other organisms

Policy information about studies involving animals; ARRIVE guidelines recommended for reporting animal research

Laboratory animals	Female 6- to 8-week-old NMRI mice (Charles River Laboratories). Housing conditions: Humidity: 57%; Temperature: 22.4 degC; Dark/Light cycle: 12 h dark/12 hrlight.
Wild animals	The study did not involve wild animals.
Field-collected samples	This study did not involve samples collected from the field.
Ethics oversight	The analysis of the fibrils was performed by ethical approval of the Regierungspräsidium Tübingen.

Note that full information on the approval of the study protocol must also be provided in the manuscript.

Human research participants

Policy information about studies involving human research participants

Population characteristics	See Methods: Extraction of amyloid fibrils from diseased tissue; Gender: Female; Age: 50 years; Diagnosis: AL amyloidosis
Recruitment	Selected based on clinical findings. Heart tissue was collected from a female patient at the age of 50, suffering from

	AL amyloidosis. The patient had cardiomyopathy as an underlying condition and underwent cardiac surgery. The case was randomly chosen out of 10 other available cases with similar pathology. This is a single case study.
Ethics oversight	The study was approved by the ethical committees of Ulm University (203/18). Informed consent was obtained from the patient for the analysis of the amyloid deposits.

Note that full information on the approval of the study protocol must also be provided in the manuscript.
